# Measuring canine impulsivity: translation and validation of a German dog impulsivity assessment scale

**DOI:** 10.3389/fvets.2026.1869578

**Published:** 2026-08-06

**Authors:** Dana Kero, Marlies Dolezal, Nadja Affenzeller

**Affiliations:** 1 Clinical Unit of Internal Medicine Small Animals, Department for Companion Animals and Horses, University of Veterinary Medicine, Vienna, Austria; 2 Platform for Bioinformatics and Biostatistics, Department of Biological Sciences and Pathobiology, University of Veterinary Medicine, Vienna, Austria

**Keywords:** DIAS, dog, factor analysis, impulsivity, psychometric questionnaire, validation

## Abstract

Psychometric questionnaires are used to assess canine temperament, aiding in the identification of behavioral and affective traits. Impulsivity, defined as a predisposition toward rapid, unplanned reactions to internal or external stimuli, is one such trait and is considered a contributing factor in various behavioral problems, including aggression. This study aimed to translate and validate the Dog Impulsivity Assessment Scale (DIAS) for use in German-speaking populations. Data from 2,788 dog–caretaker dyads were subjected to an exploratory factor analysis (EFA), revealing a four-factor structure. This structure was subsequently confirmed using a confirmatory factor analysis (CFA) in a separate subsample of 557 dyads. The resulting factors were labeled Excitability, Reactive Aggressive Behavior, Interest in Environment, and behavioral Regulation and explained 32.7% of the total variance. Three original scale items did not demonstrate adequate stability. Item 12 (“*takes a long time to lose interest*”) was removed from the final German version, whereas Items 6 (“*appears to be sorry*”) and 16 (“*reacts very quickly*”) likely reflect cross-cultural differences in interpretation. These results support the German DIAS as a useful tool for assessing impulsivity related traits in dogs and underscore the value of cross-cultural research in advancing our understanding of canine impulsivity. Further research is needed to explore cultural influences, including the increasing knowledge of dog behavior in recent decades, which may shape dog caretakers' perceptions and interpretations of their dogs' behavior and thus influence their responses to certain questions in behavioral assessment scales over time.

## 1 Introduction

Impulsivity is a personality trait that can be characterized by the speed of a reaction to internal or external stimuli during a decision-making process. Individuals with high levels of impulsivity tend to exhibit rapid, unplanned reactions without fully considering potential consequences (1). Thus, in some contexts, impulsivity is used interchangeably with self-control (2). More broadly, it reflects the degree to which an individual evaluates the outcomes of their actions (3–6). In dogs, increased impulsivity has been associated with poor decision-making, increased frustration, and the emergence of behavior problems such as excessive vocalization or aggression, particularly when coping mechanisms are underdeveloped (1).

Research indicates that impulsivity is a stable trait across time in humans, dogs, and other animal species (7–11). In dogs, impulsivity has often been studied in relation to aggressive behaviors, initially described by Fatjó et al. (12) as “impulsive aggression”; aggressive behavior that occurs with minimal or no warning signals. More recent studies suggest that impulsivity is a multifaceted trait that may contribute to broader behavioral problems. For instance, repetitive behaviors have been linked to impulsivity and may reflect deficits in behavioral regulation rather than primary emotional dysfunction (13). Impulsive dogs are thought to experience emotional states and motivations like other dogs but may struggle with behavioral inhibition (14). Similarly, Gobbo and Zupan Šemrov (2) found that dogs exhibiting high levels of aggressive reactivity often show impairments in self-control.

Psychometric tools, such as behavioral questionnaires, offer a practical and cost-effective approach to assessing temperament in dogs. These methods enable the collection of large, demographically diverse samples and are increasingly used in behavioral research (11, 15). Although experimental approaches can offer detailed behavioral insights, they are more resource intensive, and thus limited to smaller sample sizes. Both methods have strengths and limitations, and both face challenges in establishing validity and reliability (11, 16).

The Dog Impulsivity Assessment Scale (DIAS), developed by Wright et al. (17), is currently the most extensively validated psychometric instrument for assessing impulsivity in domestic dogs. This scale includes 18 items and produces an Overall Questionnaire Score (OQS), along with three subscales representing distinct components of impulsivity labeled behavioral Regulation (associated with arousal and control of responses), Aggression and Response to Novelty (relating to the display of aggressive behaviors and reactions to unfamiliar stimuli) and Responsiveness (reflecting attentiveness, trainability, and general interest in the environment) (10, 17).

The DIAS has potential applications in various settings, including behavioral research, dog training, and suitability assessments for working or assistance dogs. It may also help identify impulsivity related issues contributing to a diverse range of behavior problems and guide intervention strategies such as impulse control training (2, 18).

Although impulsivity has only recently gained attention as a distinct personality trait in dogs, its relevance is increasingly recognized, particularly in relation to aggression, inattention, and hyperactivity (19, 20).

To ensure that such instruments are applicable across different linguistic and cultural contexts, it is essential to validate translated versions to ensure that the instrument maintains its reliability, validity, and conceptual equivalence across languages. Mere linguistic translation can introduce cultural or semantic distortions, potentially compromising the accuracy of the results (21, 22). Without validation, the scale may fail to measure the intended constructs in the new context, leading to biased or invalid conclusions (23). Translation and cultural adaptation must ensure conceptual equivalence, allowing for reliable international comparison and meaningful interpretation of results in the target language. This study aimed to translate and validate the English version of the DIAS into German to enable its use in future canine temperament research and practical clinical applications.

## 2 Material and methods

### 2.1 Approval and consent

Prior to completing the online questionnaire, all dog caretakers provided informed consent to participate in the study. This research was acknowledged by the Ethics Committee, Medical University Vienna, MedUni Vienna (14/12/2018).

### 2.2 Translation

This phase involved the translation of the original 18 item Dog Impulsivity Assessment Scale [DIAS; (17)] following the World Health Organization's guidelines for instrument translation and adaptation (24). In accordance with these guidelines, the initial translation into German was followed by a back translation into English, with both steps conducted independently by bilingual translators. The back translated items were reviewed and approved by a co-author of the original scale [Daniel Mills; (17)] to ensure that the German version preserved the conceptual and semantic integrity of the original English questionnaire. This process was designed to minimize translation ambiguities and address potential semantic or cultural discrepancies that could affect item interpretation.

Subsequently, the preliminary German version was piloted with a small sample of Austrian dog caretakers (*n* = 11) to assess the clarity, comprehension, and cultural appropriateness of the items. Based on participant feedback, minor revisions were made. The final German version of the DIAS was then established through consensus among the authors (see Supplementary Table 1).

### 2.3 Questionnaire distribution

An online version of the questionnaire was developed using LimeSurvey (25). The survey was introduced under the title “What can a questionnaire tell me about my dog's character?” and included a brief description of the study's purpose and estimated completion time. Notably, the term impulsivity was deliberately omitted from the introduction to minimize response bias. The questionnaire link was disseminated via various social media platforms and promoted through physical leaflets containing a QR code, which were distributed in veterinary clinics, dog training schools, and pet stores.

Inclusion criteria required participants to be at least 18 years of age and to have lived with their dog for a minimum of 6 months. While completion of sociodemographic items was optional, all 18 items of the Dog Impulsivity Assessment Scale (DIAS) were set as mandatory within the LimeSurvey platform to ensure complete data for analysis. Data collection took place between December 2021 and March 2022.

### 2.4 Data analysis

All statistical analyses were conducted using R version 4.6.0 (26). Additonally, data processing was performed with the tidyverse package v1.3.1 (27). The distribution of individual DIAS items was examined by comparing measures of central tendency (mean, median) and dispersion (standard deviation) to assess symmetry.

The suitability of the data for factor analysis was evaluated by examining item intercorrelations. We visualized estimated pairwise polychoric correlation, calculated with function *hetcor* in package polycor v0.8-2 (28), with function corrplot.mixed in package corrplot v0.92 (29). Items were hierarchically clustered with default options. Each cell in the lower triangle of the matrix displays the estimated polychoric correlation coefficient. The circles in the upper right triangle of the matrix represent size and direction of estimated correlation coefficients.

Exploratory data analysis visualizations were generated using the ggplot2 package v3.3.5 (30).

Data cleaning involved exclusion of responses from caretakers under 18 years of age and those living with their dogs for less than 6 months. Additionally, questionnaires with more than three “not applicable” responses within the 18 DIAS items (i.e., >16.7% missing data) were removed, following criteria outlined by Savalli et al. (31).

The remaining dataset was randomly divided into two subsets (R base function sample); 5/6 of the data were allocated to exploratory factor analysis (EFA), and the remaining 1/6 was reserved for confirmatory factor analysis (CFA). EFA was performed using the psych package v2.1.9 (32). The suitability of the items for EFA was evaluated using polychoric correlation coefficients.

Factorability was assessed in both subsets via the Kaiser-Meyer-Olkin (KMO) measure of sampling adequacy and Bartlett's test of sphericity (functions KMO and cortest.bartlett in package psych v2.1.9, 32).

EFA employed maximum likelihood estimation with an oblique rotation requested with option rotate=“promax” allowing for correlations among extracted factors using function fa in R package psych v2.1.9, (32). The number of factors retained was guided by the scree plot and Kaiser's criterion (eigenvalues > 1). Items with factor loadings ≥ |0.3| were retained.

Model fit for both EFA and CFA was evaluated using the Tucker-Lewis Index (TLI) for factor reliability, the root mean square error of approximation (RMSEA), and its 90% confidence intervals.

CFA was conducted with function cfa with option *std.lv*=*TRUE* from the lavaan package v0.6-10 (33). Additionally, model fit was assessed by a Chi Square test evaluating the null hypothesis that the RMSEA is less or equal than 0.05 and Comparative Fit Index (CFI).

We further used Cronbach's alpha calculated with function alpha and McDonald's Omega Total, calculated with function omega (options: nfactors=4, poly=TRUE, fm=“ml”, rotate=“Promax”, lavaan=TRUE and for option fit providing the fitted CFA model object) both in package psych v2.1.9, (32) to assess internal consistency and report factor determinacy for CFA, extracted from the summary output of the fitted CFA model object.

Following the methodology of the original study (17), an overall impulsivity score (Overall Questionnaire Score) was calculated, alongside subfactor scores for each identified factor.

## 3 Results

### 3.1 Responses

The online survey link was accessed 4255 times. Of these, 3355 met the inclusion criteria (≥18 years; dog ownership ≥6 months). After excluding cases with ≥4 “not applicable” responses in the DIAS, the final sample comprised 3,345.

### 3.2 DIAS items

Item 6 (“*appears to be sorry”*) received the highest proportion of “not applicable” responses (5.42%). All other items had “not applicable” response rates below 5%. These responses were excluded from further analysis.

Four items (Items 2, 5, 9, and 18) displayed substantial negative skewness, with means below 2 and medians of 1, indicating a strong tendency toward lower response values. These items included two related to aggressive behaviors (Items 5 and 9) and two related to excitability (i.e., “*occurrence of repetitive behavior when excited”* and “*becoming excited for no reason”*). In contrast, Item 15 (“*interested in new things and new places”*) was positively skewed (mean > 4, median = 5), reflecting a high frequency of dogs rated as highly exploratory or curious. Despite these distributional deviations, all five items were retained in the EFA, as they represent core components of the impulsivity construct.

### 3.3 Details exploratory factor analysis

Given the large number of fully completed questionnaires and the known sensitivity of fit indices to large sample sizes, the data were not divided into two equal subsets, as suggested in previous studies (e.g., 31). Instead, a larger randomly sampled subsample (*n* = 2,788) was used to conduct the EFA, while a smaller subsample (*n* = 557) was reserved for CFA. The CFA sample size closely aligns with that used in the original DIAS validation study (*n* = 560; 17) and is therefore considered sufficient for model confirmation.

To ensure comparability between the EFA and CFA subsamples, descriptive statistics were calculated for each. No significant differences were observed in these parameters, indicating that the two subsamples were statistically comparable and representative of the overall dataset.

#### 3.3.1 Sampling and item adequacy

**Figure 1:**
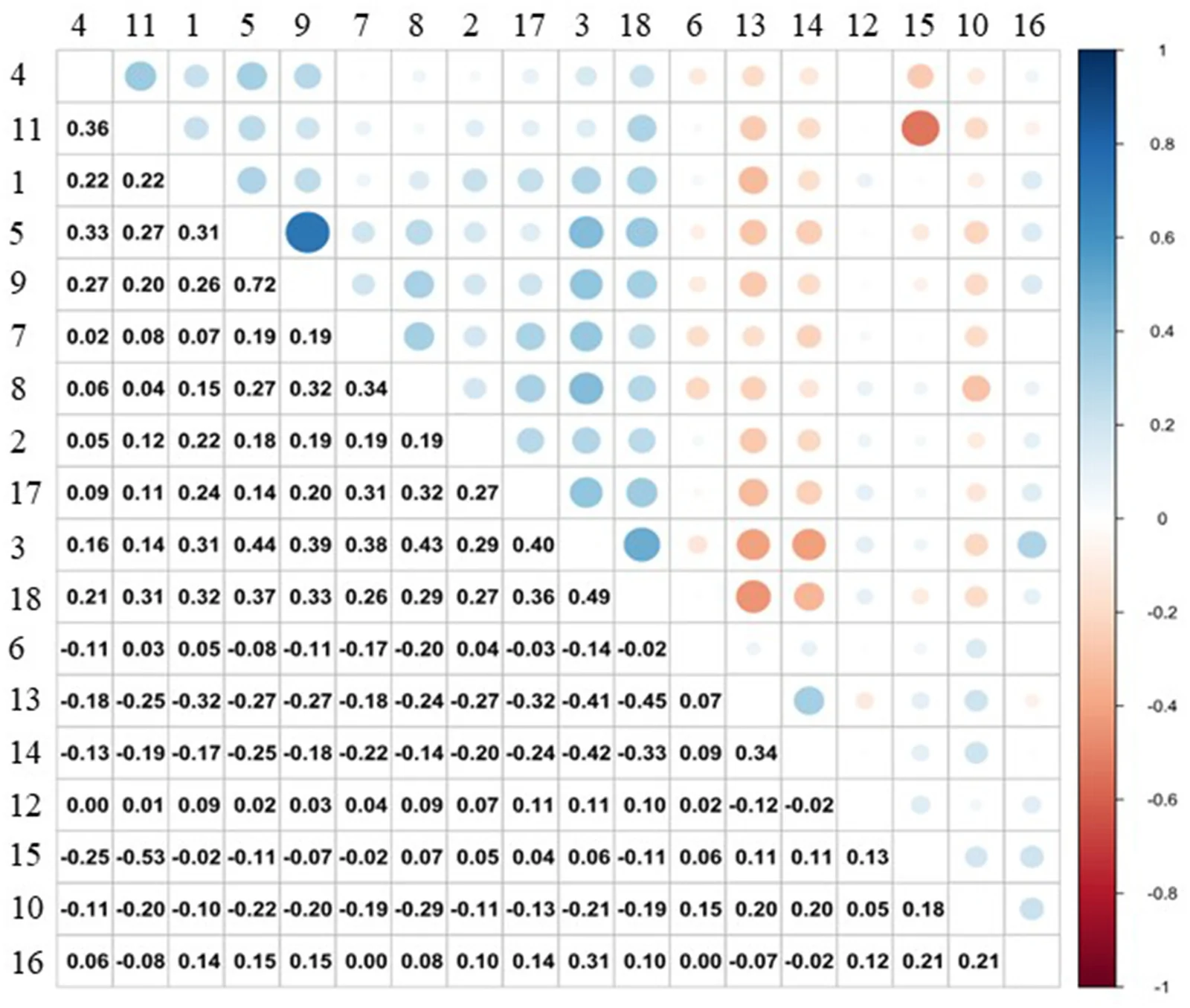
Correlation plot of all pairwise polychoric correlation coefficients. All numerical values are presented in the lower left triangle of the matrix. Dot size demonstrates the graphical representation of the numerical value in the upper right triangle. Blue dots represent a positive correlation, whereas red dots display a negative correlation.

The suitability of the items for EFA was first evaluated using polychoric correlation coefficients. A strong positive correlation (*r* = 0.72) was observed between Item 5 and Item 9, both of which assess aggressive responses triggered by frustration and arousal. Item 11 (“*not keen to go into new situations”*) and Item 15 (“*interested in new things and new places”)* were negatively associated (*r* = −0.53). All other item correlations were below 0.5 (see Figure 1). As expected, Item 3 (“*dog is impulsive”*) exhibited the highest number of moderate correlations with other items. These included associations with aggression related items (Item 5: *r* = 0.44; Item 9: *r* = 0.39), excitability (Item 18: *r* = 0.49), persistence (Item 8: *r* = 0.43), impatience (Item 17: *r* = 0.40), and acting without thinking (Item 7: *r* = 0.38). Additionally, Item 3 was moderately negatively correlated with self-regulation items, such as Item 14 (“*has a lot of control”, r* = −0.42) and Item 13 (“*calms down quickly”, r* = −0.34).

No correlations exceeded *r* = 0.80, indicating the absence of multicollinearity; therefore, no items were considered redundant.

Further confirmation of the data's suitability for EFA was provided by a KMO coefficient of 0.82, with all 18 items yielding individual measures of sampling adequacy (MSA) greater than 0.60; range 0.62–0.91). Bartlett's test of sphericity was statistically significant (χ^2^ = 12,247.23, *df* = 153, *p* < 0.001), and the determinant of the polychoric correlation matrix was 0.012, indicating no evidence of singularity or relevant multicollinearity, supporting the appropriateness of factor analysis. Consequently, all 18 items were retained for the EFA.

#### 3.3.2 Factor analysis and loadings

**Figure 2:**
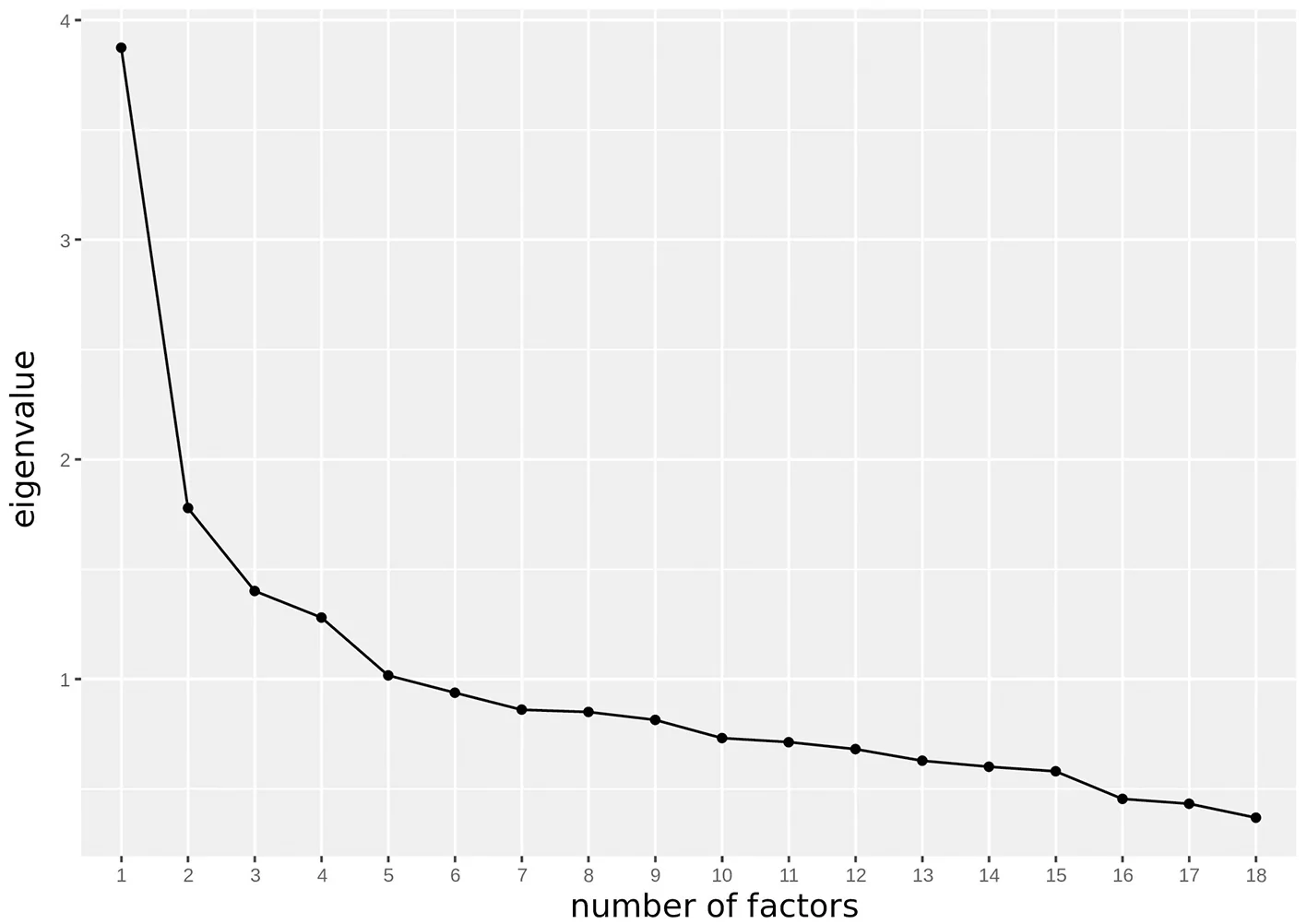
Eigenvalue distribution of the items of the exploratory factor analysis (EFA) in a scree plot. The x-axis represents the 18 items and, correspondingly, the possible number of factors, while the y-axis displays the eigenvalues associated with each item. A noticeable flattening of the curve occurs around the fifth item, indicating a four-factor solution as the most suitable (34). Consistent with this interpretation, the first four factors exhibited eigenvalues greater than 1, in line with the Kaiser criterion.

Horn's parallel analysis (34), in conjunction with the Scree plot (35), supported the extraction of four factors based on Kaiser's eigenvalue criterion (eigenvalues > 1, Figure 2). Both methods yielded consistent results, indicating a four-factor solution as the most appropriate structure for the data [Figure 2; (36)].

Using the maximum likelihood extraction method, the four identified factors accounted for 32.7% of the total variance. Specifically, Factor 1 explained 10.9%, Factor 2 explained 7.9%, Factor 3 explained 7.6%, and Factor 4 explained 6.2% of the variance (see Table 1). Given the relatively even contribution of each factor and alignment with clinical knowledge and experience, the four-factor model was retained.

**Table 1:** Variance values of the exploratory factor analysis (EFA).

Variance values	Factor 1	Factor 2	Factor 3	Factor 4
Sum of sqared factor loadings	1.964	1.426	1.368	1.123
Proportions of variance	0.109	0.079	0.076	0.062
Cumulative proportions of variance	0.109	0.188	0.264	0.327
Explained proportions of variance	0.334	0.243	0.233	0.191
Cumulative proportions of explained variance	0.334	0.577	0.809	1.000

The sum of squared factor loadings corresponds to the eigenvalue of each factor after rotation. The proportions of variance indicate the share of total variance explained by each factor. The cumulative proportions of variance represent the total variance explained up to the given factor. The explained proportions of variance express the variance accounted for by each factor relative to the total explained variance. The cumulative proportion of explained variance refers to these explained proportions and represents their cumulative sum across factors.

Promax-rotated factor loadings are presented in Table 2.

**Table 2:** Promax rotated factor loadings of the exploratory factor analysis (EFA).

Item	Factor 1	Factor 2	Factor 3	Factor 4	*h* ^2^	*u* ^2^
1	**0.429**	0.091	−0.120	−0.136	0.2373	0.763
2	***0.339***	−0.031	0.010	0.049	0.1156	0.884
3	**0.557**	0.133	0.100	0.289	0.5700	0.430
4	0.084	0.214	−***0.316***	−0.090	0.1998	0.800
5	−0.092	**0.861**	−0.001	0.087	0.7053	0.295
6	0.079	−0.042	−0.065	−***0.348***	0.1061	0.894
7	0.196	−0.040	0.071	**0.463**	0.2763	0.724
8	0.177	0.102	0.150	**0.492**	0.3384	0.662
9	−0.081	**0.735**	0.059	0.151	0.5248	0.475
10	0.052	0.041	0.176	−**0.411**	0.2415	0.759
11	0.181	−0.012	−**0.746**	−0.150	0.5661	0.434
12	0.281	−0.054	0.076	−0.075	0.0658	0.934
13	−**0.505**	0.020	0.187	−0.044	0.3284	0.672
14	−**0.358**	0.027	0.141	−0.161	0.2259	0.774
15	0.127	0.077	**0.722**	0.031	0.4795	0.520
16	***0.343***	0.142	0.245	−0.195	0.2108	0.789
17	**0.523**	−0.134	0.035	0.203	0.3075	0.692
18	**0.498**	0.055	−0.182	0.068	0.3821	0.618

h^2^, communalities; u^2^, unexplained variance; values in bold represent factor loadings reaching a cut off |≥.35|; bold italicized values represent factor loadings reaching a cut off |≥.30|.

Factor 1 (F1, Items 1, 2, 3, 13, 14, 16, 17, 18) was interpreted as *Excitability*, encompassing items related to arousal, lack of patience, and emotional intensity.

Factor 2 (F2, Items 5 and 9) was labeled *Reactive Aggressive Behavior*, capturing behaviors associated with frustration and arousal induced aggressive behavioral responses.

Factor 3 (F3, Items 4, 11, 15) was interpreted as *Interest in Environment*, reflecting attentiveness and curiosity toward novel stimuli.

Factor 4 (F4, Items 6, 7, 8, 10) was labeled behavioral *Regulation*, including items related to persistence, impulsive action, and self-control.

Item 12 did not load on any factor above the threshold of |≥.30| and was excluded from the factor interpretation.

#### 3.3.3 Fit of the model and reliability of measures

Model fit indices indicated an adequate representation of the data, with a TLI of 0.906 a RMSEA of 0.044 (90% CI [0.041, 0.048]) and RMSR = 0.026. These values meet conventional criteria for a good fit (TLI ≥ 0.90, RMSEA < 0.05; (37, 38)). The 90% confidence interval for the RMSEA ranged from 0.041 to 0.048, remaining below the threshold of 0.05, further supporting the adequacy of the model.

For the four factor EFA solution, factor score adequacy was strong; correlations of regression scores with factors were F1 0.77, F2 0.82, F3 0.83, and F4 0.71. The corresponding multiple R square values were F1 0.59, F2 0.68, F3 0.69, and F4 0.50.

Reliability measures for internal consistency of factor score adequacy were acceptable (Cronbach's α = 0.76; 95% CI [0.75, 0.77]; Mc Donald ω total = 0.81).

#### 3.3.4 Factor intercorrelations

Factor intercorrelations analysis revealed significant correlations among the four factors. The strongest correlation was observed between Factor 1 (Excitability) and Factor 2 (Reactive Aggressive Behavior) *r* = 0.51, followed by the correlation between Factor 1 (Excitability) and Factor 4 (behavioral Regulation) with *r* = 0.31 and Factor 2 (Reactive Aggressive Behavior) and Factor 3 (Interest in Environment) with *r* = −0.31. The weakest correlation was found between Factor 3 and Factor 4 with *r* = −0.30.

### 3.4 Details confirmatory factor analysis

The suitability of the data for CFA was supported by a KMO value of 0.78 with item MSAs ranging from 0.60 to 0.89 and a significant Bartlett's test of sphericity (χ^2^ = 2,619.188, *df* = 153, *p* < 0.001). The determinant of the correlation matrix was 0.009, confirming adequate sampling adequacy and correlation structure within the subsample.

**Figure 3:**
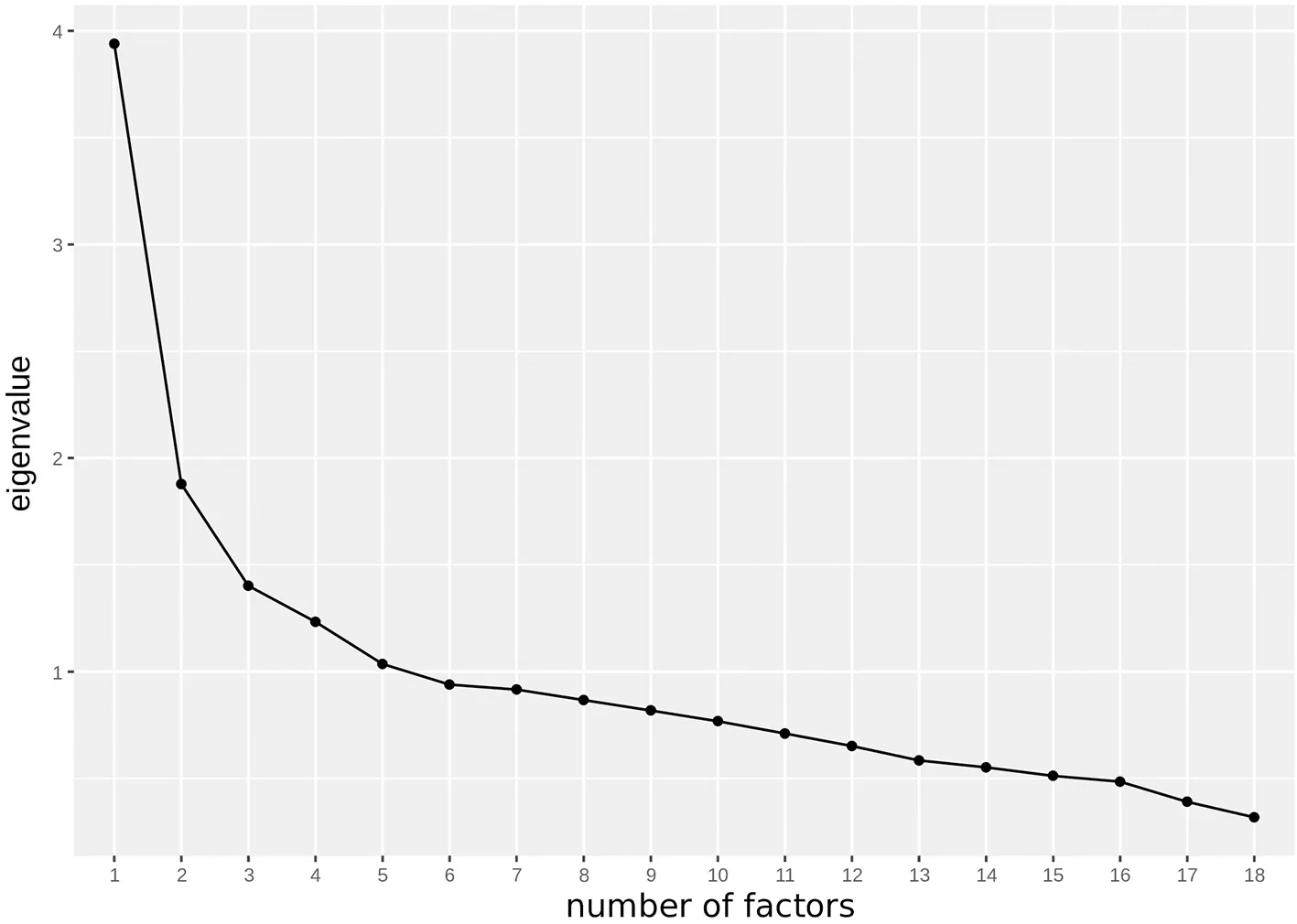
Eigenvalue distribution of the items of the confirmatory factor analysis (CFA) in a scree plot. The x-axis represents the 18 items and, correspondingly, the possible number of factors, while the y-axis displays the eigenvalues associated with each item. A noticeable flattening of the curve occurs around the fifth item, indicating a four-factor solution as the most suitable (34). Consistent with this interpretation, the first four factors exhibited eigenvalues greater than 1, in line with the Kaiser criterion.

Consistent with EFA results, both Horn's parallel analysis (34) and Cattell's scree test (35) supported a four-factor solution (Figure 3). Item loadings (*r* |≥.30|) were again aligned with the same four factors. However, Item 12 did not load significantly on any factor.

**Figure 4:**
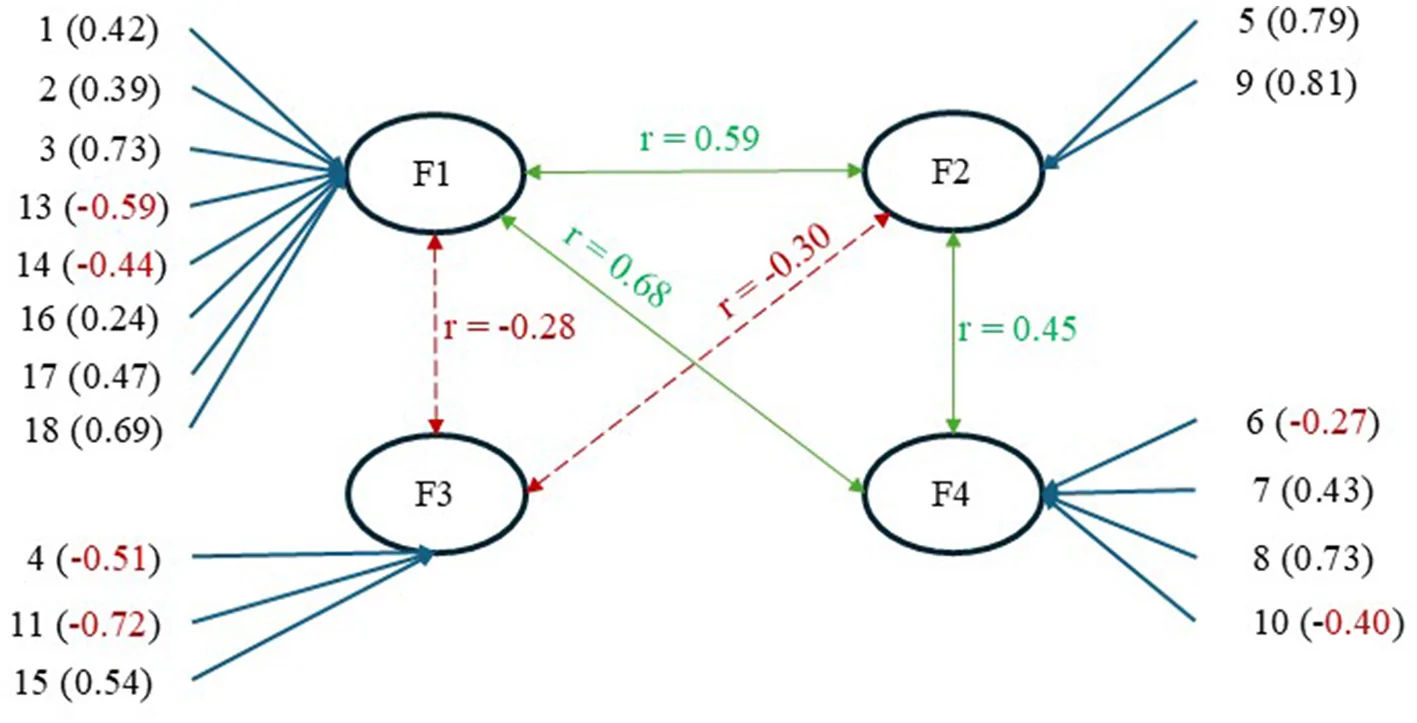
Confirmatory factor analysis (CFA) path diagram and interfactor correlations. All four latent factors (F1–F4) and their observed items (1-18) Standardized factor loadings are shown in parentheses next to each item. Double headed arrows represent correlations between latent factors with the corresponding correlation coefficient (r) displayed next to the arrows. Positive correlations are shown in green and negative correlations in red-dashed. All displayed correlations were statistically significant (*p* < 0.001).

Standardized loadings were consistent with the EFA structure: Factor 1 (items 1, 2, 3, 13, 14, 16, 17, 18) |λ| = 0.24–0.73, with item16, λ = 0.24 not loading adequately; Factor 2 (items 5,9) |λ| = 0.79–0.81; Factor 3 (items 4, 11, 15); |λ| = 0.51–0.72; Factor 4 (items 6, 7, 8, 10) |λ| = 0.27–0.73 with item 6, λ = 0.24 not loading adequately. For details see Figure 4.

The retained items and final factor structure are presented in Tables 3, 4.

**Table 3:** Final factor structure, retained items and standardized solution of the model.

Identified factors	Item	Standardized factor loadings	Systematic proportion of variance
Excitability (factor 1)	(1)	0.424	17.9%
	(2)	0.385	14.8%
	(3)	0.734	53.8%
	(13)	−0.588	34.6%
	(14)	−0.442	19.5%
	(16)	0.239	5.7%
	(17)	0.465	21.7%
	(18)	0.689	47.5%
Reactive aggressive behavior (factor 2)	(5)	0.787	61.9%
	(9)	0.807	65.2%
Interest in environment (factor 3)	(4)	−0.501	26.0%
	(11)	−0.723	52.3%
	(15)	0.543	29.5%
Behavioral Regulation (factor 4)	(6)	−0.268	7.2%
	(7)	0.431	18.6%
	(8)	0.727	52.9%
	(10)	−0.397	15.7%

Standardized factor loadings indicate the strength and direction of the relationship between each item and its factor; systematic proportion of variance indicates how much of each items' variance is explained by the factor.

**Table 4:** Final factor solution for the 17 German DIAS items, including the biological interpretation of the factors.

Factor 1	Factor 2	Factor 3	Factor 4
(1) My dog shows extreme physical signs when excited (e.g., drooling, panting, raising hackles, urination, licking lips, widening of eyes).	(5) My dog becomes aggressive (e.g., growl, snarl, snap, bite) when excited.	(4) My dog doesn't like to be approached or hugged.	(6) My dog appears to be ‘sorry' after it has done something wrong.
(2) When my dog gets very excited it can lead to fixed repetitive behavior (i.e., an action that is repeated in the same way over and over again), such as tail chasing or spinning around in circles.	(9) My dog may become aggressive (e.g., growl, snarl, snap, bite) if frustrated with something.	(11) My dog is not keen to go into new situations.	(7) My dog does not think before it acts (e.g., would steal food without first looking to see if someone is watching).
(3) I would consider my dog to be very impulsive (i.e., has sudden, strong urges to act; acts without forethought; acts without considering effects of actions).	–	(15) My dog is very interested in new things and new places.	(8) My dog can be very persistent (e.g., will continue to do something even if it knows it will get punished or told off).
(13) My dog calms down very quickly after being excited	–	–	(10) My dog is easy to train.
(14) My dog appears to have a lot of control over how it responds.	–	–	–
(16) My dog reacts very quickly.	–	–	–
(17) My dog is not very patient (e.g., gets agitated waiting for its food, or waiting to go out for a walk).	–	–	–
15.6-7.4,-1.3498pt(18) My dog seems to get excited for no reason.	–	–	–
Biological interpretation			
Excitability	Reactive aggressive behavior	Interest in environment	Behavioral regulation

#### 3.4.1 Fit of the model and reliability of measures

Model fit statistics from the CFA indicated an overall acceptable fit; test statistic χ^2^(113) = 318.23, *p* < 0.001; CFI = 0.868; TLI = 0.841; RMSEA = 0.062 (90% CI [0.054–0.070]); Standardized Root Mean Square Residual = 0.060.

For the four factor CFA solution, factor score adequacy was acceptable; correlations of regression scores with factors were F1 0.81, F2 0.67, F3 0.83, and F4 0.75. The corresponding multiple R square values were F1 0.65, F2 0.44, F3 0.69, and F4 0.56.

Reliability measures for internal consistency of factor score adequacy were acceptable (Cronbach's α = 0.76; 95% CI [0.73–0.79]; Mc Donald ω total = 0.85).

Additionally, the CFI value was 0.87, approaching the conventional 0.9 threshold supporting acceptable model fit, particularly in the context of a large sample (37).

#### 3.4.2 Factors and contributing items

To assess the relative contribution of each item to its corresponding factor, the proportion of systematic variance was calculated (for details see Table 3). For an overview the two highest loading items per factor and their respective variance contributions are presented.

Factor 1: Excitability (total of 8 items):

- Item 3 (“*is impulsive”*) contributed 53.8%.
- Item 18 (“*becomes excited for no reason”*) accounted for 47.5% of the systematic variance.

Factor 2: Reactive Aggressive Behavior (total of 2 items):

- Item 5 (“*aggressive when excited”*) and Item 9 (“*aggressive when frustrated”*) both showed strong loadings (r ≈ 0.8), explaining 61.9% and 65.2% of the variance, respectively.

Factor 3: Interest in Environment (total of 3 items):

- Item 11 (“*not keen to go into new situations”*) explained 52.3% of the variance.
- Item 15 (“*interested in new things and new places”*) contributed 29.5%.

Factor 4: behavioral Regulation (total of 4 items):

- Item 8 (“*being persistent”*) explained 52.9% of the variance.
- Item 7 (“*not thinking before acting”*) accounted for 18.6%.

Covariance analysis revealed significant correlations among all four factors. The strongest correlation was observed between Factor 1 (Excitability) and Factor 4 (behavioral Regulation) *r* = 0.68, followed by the correlation between Factor 1 (Excitability) and Factor 2 (reactive aggressive behavior) with *r* = 0.59. Factor 2 (reactive aggressive behavior) also showed a moderate correlation with Factor 4 (behavioral regulation) *r* = 0.45. Factor 3 (Interest in Environment) demonstrated the weakest correlations with the other factors: *r* = −0.28 with Factor 1, *r* = −0.30 with Factor 2, and *r* = −0.12 with Factor 4.

Standardized scale scores were calculated for the overall impulsivity score and each of the four subscales. The mean Overall Questionnaire Score (OQS) was 0.53 (SD ± 0.1; mean ± SD 0.43–0.63) Factor 1 (excitability) had a mean score of 0.52 (SD ± 0.13; mean ± SD 0.39–0.65). Factor 2 (reactive aggressive behavior) showed a mean score of 0.33 (SD ±0.18; mean ± SD 0.15–0.51). Factor 3 (interest in environment) had the highest mean score of 0.68 (SD ± 0.13; mean ± SD 0.55–0.81). Factor 4 (behavioral regulation) had a mean score of 0.52 (SD ± 0.13; mean ± SD 0.39–0.65).

### 3.5 Sociodemographic profiles

#### 3.5.1 Caretaker characteristics

Of the 3,345 completed questionnaires, the majority were submitted by female respondents (*n* = 3,115; 93.1%), with male participants comprising 6.2% (*n* = 208) of the sample. The mean age of respondents was 39.9 years. Age distribution was as follows: 18–30 years (*n* = 908; 27.1%), 31–45 years (*n* = 1,321; 39.5%), 46–60 years (*n* = 928; 27.7%), and over 60 years (*n* = 188; 5.6%). Participants were primarily from Austria (47.7%) and Germany (45.1%), with smaller proportions from Switzerland (4.7%) and other countries (2.4%). When asked whether their dog exhibited behavioral problems, 30.0% of respondents (*n* = 1,005) reported that they did, while 69.6% (*n* = 2,327) did not report any behavioral concerns.

#### 3.5.2 Dog characteristics

The dog sample consisted of 2,000 pedigree dogs (59.8%), 1,305 mixed-breed dogs (39.0%) and 40 of unknown pedigree (0.2%). The 10 most frequently represented breeds included: Australian Shepherd (*n* = 186; 9.3%), Labrador Retriever (*n* = 163; 8.2%), Border Collie (*n* = 105; 5.3%), German Shepherd (*n* = 71; 3.6%), Golden Retriever (*n* = 69; 3.5%), Belgian Shepherd (*n* = 44; 2.2%), Jack Russell Terrier (*n* = 40; 2.0%), Rhodesian Ridgeback (*n* = 40; 2.0%), Chihuahua (*n* = 37; 1.9%) and Poodle (*n* = 36; 1.8%).

The median age of the dogs was 5.0 years (IQR 3.0–8.5). The average body weight was 22.1 kg, with distribution across size categories as follows: very small (< 5 kg): *n* = 104 (3.1%), small (5–10 kg): *n* = 482 (14.4%), medium (10.5–25 kg): *n* = 1,535 (45.9%), large (25.5–45 kg): *n* = 1,119 (33.5%), very large (>45 kg): *n* = 104 (3.1%).

The sex distribution of the dogs was balanced, with 1,702 females (50.9%) and 1,637 males (48.9%). A slight majority of the dogs were neutered (*n* = 1,862; 55.7%), with the remaining 43.7% intact.

With respect to origin, almost half of the dogs (*n* = 1,646; 49.2%) were obtained from breeders. Additionally, 1,057 dogs (31.6%) came from animal shelters or welfare organizations, 332 (9.9%) from acquaintances (e.g., friends, neighbors, relatives), 85 (2.5%) from private organizations, and 67 dogs (2.0%) were bred by their current caretakers.

## 4 Discussion

This study aimed to translate and validate the original English DIAS, a psychometric questionnaire on canine impulsivity into German to enable its use in future clinical applications and canine temperament research.

The current study identified a four-factor structure through exploratory and confirmatory factor analyses, differing from the original three factor model proposed in the DIAS questionnaire by Wright et al. (17). Specifically, the data were best explained by four distinct dimensions labeled as: *Excitability, Reactive Aggressive Behavior, Interest in the Environment* and *behavioral Regulation*. This divergence is likely due to the substantially larger sample size in the present study (*n* = 3,345 vs. 560), which enhanced statistical power and enabled the detection of more nuanced behavioral constructs. In addition, potential translation-related effects, differences in respondent populations and cultural influences may also have affected responses and contributed to discrepancies between the present study and the original DIAS. Also, methodological choices such as recruitment strategies and data collection may have influenced the results. Notably, the original second factor (“Aggression and Response to Novelty”) appeared to fractionate into two separate factors: *Reactive Aggressive Behavior* and *Interest in the Environment*.

In addition, one (Item 12 “*takes long to lose interest*”) was excluded from the German version. Importantly, the variance was more evenly distributed across the four factors, suggesting improved resolution and robustness of this factor structure and the underlying behavioral dimensions due to the larger dataset.

### 4.1 Factor 1 excitability

Factor 1, labeled *Excitability*, comprised 8 items (Items 1, 2, 3, 13, 14, 16, 17, 18) reflecting states of heightened arousal, impatience, difficulty in calming down, and emotional intensity. Notably, all 7 items loaded onto the behavioral Regulation factor in the original questionnaire, with Items 13 “*calms down quickly*” and Item 14 “*has a lot of control*” showing negative loadings, consistent with the original scale structure. In contrast, items 7 (“*not thinking before acting*”), 8 (“*being persistent*”), and 10 (“*easy to train*”; loading negatively), which also belonged to the original behavioral Regulation factor, emerged as a distinct factor in the present study, leading us to label Factor 4 as behavioral Regulation.

In this study Factor 1 “*Excitability*” refers to an individual's predisposition to heightened emotional and physiological arousal, accompanied by impatience, restless and repetitive behaviors and in general dogs getting agitated easily. High excitability is marked by low thresholds for arousal and difficulty calming down after stimulation. Studies in humans and dogs confirm that arousal plays a significant role in the expression of impulsivity (39). In addition, disobedience, destructiveness and barking behavior where amongst the most often reported behavior problems by dog caretakers, who classified their dog as “excitable” (40, 41).

It is particularly interesting that, when analyzing various validated questionnaires from dog caretakers who completed all of them for the same dogs, the subcategory “excitability” of the canine behavioral assessment and research score (CBARQ) showed a high correlation (*r* = 0.49) with Factor 1 of the original DIAS (19), which supports the interpretation of this study. Further studies may shed light on the relationship between impulsivity, excitable behaviors in dogs and measures of physiological arousal.

### 4.2 Factor 2 reactive aggressive behavior

In human psychology, impulsive aggression is characterized by sudden, emotionally driven aggressive outbursts in response to perceived provocation or frustration. It is closely associated with trait impulsivity, a predisposition to act with reduced forethought, poor behavioral inhibition, and heightened emotional reactivity (4, 42). This form of aggression is typically reactive, defensive, and affectively charged, often arising in individuals with dysregulated emotion regulation systems, including altered functioning in the prefrontal cortex and amygdala (43).

In the study of canine behavior, parallels have been drawn between human impulsive aggression and certain forms of aggressive behaviors in dogs, particularly those that are sudden and appear poorly modulated. Dogs exhibiting these behaviors often demonstrate heightened arousal, low thresholds for reaction, and reduced behavioral inhibition, consistent with elevated impulsivity as a trait (2, 12). Behavioral assessments and caretaker reports have supported the existence of individual variation in canine impulsivity, influencing reactions and responses to environmental stimuli and aggression thresholds (10, 44, 45).

Referring to dogs' behavior as “impulsive aggression” poses conceptual difficulties. In human clinical contexts, this label encompasses a complex interaction of cognitive and emotional processes that are not readily observable or measurable in non-human animals. Therefore, we suggest using the term “*reactive aggressive behavior*” to more accurately and cautiously describe Factor 2, which includes Item 5 (“*aggressive when excited*”) and Item 9 (“*aggressive when frustrated*”). This terminology better reflects sudden aggressive behaviors in dogs that may arise in response to perceived threats or frustration and are characterized by heightened emotional arousal and impaired behavioral inhibition.

Importantly, reactive aggressive behavior should be interpreted within the broader framework of canine impulsivity rather than as a distinct diagnostic category. Questionnaire scores alone cannot determine the underlying motivation, emotional state, or cognitive processes associated with aggressive behavior. Consequently, assessment of canine impulsivity should incorporate a comprehensive clinical and behavioral evaluation of the individual dog, including its history, environment, and the Overall Questionnaire Score (OQS) and its subdomains. Such an approach helps avoid prematurely labeling a dog as “aggressive” based solely on questionnaire results.

Although the emergence of a factor reflecting reactive aggressive behavior is of importance, this factor comprised only two items and should therefore be interpreted with caution. Two-item factors may be less stable psychometrically and can also reflect item-specific covariance rather than a robust latent construct. Consequently, the present findings should be considered preliminary and require replication in independent populations before firm conclusions can be drawn regarding the existence of a distinct impulsivity related dimension.

Notably, Factor 2 (“Aggression and Response to Novelty”) of the original DIAS was found to correlate with stranger directed (*r* = 0.32, *p* < 0.001) and dog directed (*r* = 0.32, *p* < 0.001) aggression on the CBARQ (19), highlighting the distinct origins and different types of aggressive behaviors in dogs.

### 4.3 Factor 3 interest in environment

Factor 3 comprised of 3 items (4 “*does not like approach*”, 11 “*not keen to go into new situations”*, 15 “*interested in new things and new places*”) of which both items 4 and 11 were loading negatively. This factor had the highest mean score of 0.68 and was interpreted as inquisitive, exploratory and positive social behavior.

Factor 2 (“Aggression and Response to Novelty”) in the original DIAS correlated to stranger directed (*r* = 0.49, *p* < 0.001), non-social (*r* = 0.31, *p* < 0.001) and dog directed fear (*r* = 0.36, *p* < 0,001) in the CBARQ (19). Given these correlations, a clinical interpretation of a below mean score in a dog might indicate fear and anxiety related tendencies, that might warrant further exploration and assessment by a behavior expert. In contrast, a dog scoring above average can be viewed as impulsively moving into new situations without fully assessing those situations beforehand; a classical feature of human impulsive behavior, often classified as sensation seeking or risk-taking behavior, a tendency to pursue exciting activities and an openness to engaging in new experiences (46–48). However, these constructs were not directly assessed in the present study. Therefore, this interpretation should be considered a hypothesis for future investigation rather than a conclusion supported by the current data.

### 4.4 Factor 4 behavioral regulation

Factor 4 is defined by four items (Item 6 “*(“appears to be sorry”*”, Item 7 “*not thinking before acting*”, Item 8 “*being persistent*” and Item 10 “*easy to train*” loading negatively). Together, these items reflect aspects of behavioral persistence, acting without fully considering the consequences and difficulties in trainability.

Interestingly, when the original DIAS was used to assess differences in trait impulsivity between working line and show line dogs of two breeds, Item 10 also loaded on a different factor (Factor 3 “Responsiveness”) (11). This was interpreted as reflecting the requirement for working gundogs to demonstrate high trainability to remain responsive and obedient for prolonged periods during work (49). In the present analysis, the negative loading of Item 10 (“*easy to train*”) on behavioral Regulation appears consistent with this interpretation, as dogs that are more difficult to train would be expected to exhibit greater difficulties in behavioral regulation. Consequently, lower scores on trainability align with higher levels of impulsivity and poorer behavioral control.

The strongest correlation in the covariance analysis was observed between Factor 1 and Factor 4 (*r* = 0.68). The relatively strong correlation between “Excitability” and “behavioral Regulation” suggests that these dimensions likely reflect interrelated aspects of impulsivity in dogs. The substantial shared variance between these factors is consistent with the notion that they represent related facets of a broader construct of canine impulsivity, as captured by the Overall Questionnaire Score (OQS). Nevertheless, the CFA supported retaining both factors as distinct but correlated latent factors, suggesting that they capture separable facets of impulsivity. Accordingly, the OQS and subdomain scores can provide complementary information, with the former reflecting overall impulsivity and the latter offering insight into specific behavioral manifestations of the construct.

Factor 4 (behavioral Regulation) in the present study shows comparable mean values to the correspondingly named factor in the original DIAS. Higher scores indicate greater difficulties in behavioral regulation and thus higher levels of impulsivity, whereas lower scores indicate dogs with greater emotional stability.

### 4.5 Items 6, 12 and 16

Increased knowledge of canine behavior over the past decade likely explains why Item 6 (“*appears to be sorry*”) had the highest “not applicable” responses (5.42%). This item did not significantly load (*r*= |≥.30| in the CFA on any of the 4 factors, consistent with findings by Fadel et al. (11), who replicated the DIAS structure in her studies on trait impulsivity across different working and show dog breeds. Indeed, feedback from dog caretakers and canine researchers familiar with the tool suggested that this question is interpreted differently depending on the respondent's knowledge of dog behavior. Recent studies indicate that the “guilty look” expression in dogs is a direct reaction to the caretaker's behavior rather than an indication of the dog's awareness of wrongdoing (50–52). This evolving understanding highlights how earlier anthropomorphic assumptions embedded in the original questionnaire may lead to ambiguity of terms like “feeling guilty.” Consequently, validation (and potentially even revalidation) of canine temperament questionnaires is essential to ensure accurate replication of scale structures and psychometric properties over time and evolving knowledge on canine behavior. Alongside the influence of public knowledge of canine behavior there are also additional aspects that should be considered as potential influence on why some of the items failed to load adequately. Such considerations include cultural differences, semantic ambiguity and differences in respondent populations as well as translational effects.

In the original DIAS questionnaire, both Item 12 (“*takes long to lose interest*”) and Item 16 (“*reacts very quickly*”) were assigned to Factor 3, labeled ‘Responsiveness,' which reflects general environmental awareness and responsiveness, where higher scores indicate greater trainability, sustained interest in stimuli, and fast reactions. However, in the current study, item 12 did not load in the EFA and was therefore excluded from further analysis. Item 16 loaded non- significantly (*r* = 0.24) on factor one.

We propose that imprecise phrases such as “long time” and “very quickly” may have contributed to the observed inconsistencies, highlighting the difficulties of using ambiguous language without operational definitions when assessing psychometric tools (53, 54). While we attempted to minimize this bias by piloting the initial German version with a small group of Austrian dog caretakers to evaluate item clarity, comprehension, and cultural relevance, we propose that future research should involve operational definitions.

### 4.6 Appropriateness of the data supporting a 4-factor structure

The dataset demonstrated strong suitability for EFA with a high KMO measure (0.82) indicating appropriate sampling adequacy, and low polychoric correlation coefficients (all < 0.8), confirming the absence of multicollinearity among the 18 German-language items. A significant Bartlett's test of sphericity and the determinant of the correlation matrix further supported the data suitability for factor analysis.

Horn's parallel analysis (34), in conjunction with the Scree plot (35), supported the extraction of four factors based on Kaiser's eigenvalue criterion (eigenvalues > 1). Both methods yielded consistent results, indicating a 4-factor solution as the most appropriate structure for the data. Model fit indices for the EFA (TLI and RMSEA) confirmed this structure, both indices meeting established benchmarks for good model fit (TLI > 0.90; RMSEA < 0.05, (37, 38)). The four extracted factors collectively explained 32.7% of the total variance. The relatively balanced variance contributions and alignment with clinical expectations justified retaining the four-factor solution for subsequent CFA. The data were likewise suitable for CFA. The KMO value of 0.78 and a statistically significant Bartlett's test of sphericity (*p* < 0.001) confirmed sampling adequacy and the presence of sufficient correlations among items. The determinant of the correlation matrix further supported the appropriateness of factor analysis. Consistent with EFA findings, both Horn's parallel analysis and Cattell's scree test (35) reinforced the 4-factor structure as the most meaningful solution. This discrepancy from the original DIAS findings is probably attributable to the much larger sample in the current study (*n* = 3,345 vs. 560), which provided greater statistical power and allowed more subtle behavioral patterns to be identified. Nevertheless, as noted earlier, additional research is required to examine cultural factors, such as the growing public understanding of dog behavior in recent decades, that may influence how caregivers perceive and interpret their dogs' actions, ultimately affecting their responses to items on behavioral assessment scales over time.

### 4.7 Caretaker reported data and item retention considerations

This study was based on caretaker-reported assessments, which inherently carry the risk of social desirability bias (55). Such bias may lead to inflated responses on positively framed items and underreporting on items associated with undesirable behaviors. While conventional psychometric guidelines recommend excluding items with extreme response distributions due to potential limitations in variability and reliability, four items were retained in this study due to their theoretical and clinical relevance to impulsivity in dogs.

Specifically, behavioral domains such as displays of aggressive behaviors (Items 5 and 9), repetitive behaviors (Item 2), and unprovoked arousal (Item 9) were preserved in the analysis. These domains are well established in the scientific literature as key features of impulsivity related phenotypes [e.g., (1, 2, 12, 13, 19, 58)]. Despite exhibiting negatively skewed response distributions, their exclusion would have compromised the construct validity of the scale by omitting clinically relevant and meaningful behaviors.

Similarly, the positively skewed item 15 (“*interested in new things and new places*”) was retained based on the conceptual importance in capturing dimensions of attention and approach tendencies, traits closely linked to impulsive behavior. While such items may not meet all statistical inclusion criteria, their theoretical justification warranted their inclusion to ensure comprehensive coverage of the impulsivity construct (56).

Overall, the reliance on caretaker reported data necessitates cautious interpretation of results. The influence of social desirability bias likely contributed to the observed distribution patterns, with lower scores on items associated with problematic behaviors and elevated scores on more favorable traits. Nonetheless, the inclusion of these items, despite statistical skewness, was essential to preserve the integrity of the impulsivity construct being measured.

### 4.8 Motor vs. cognitive impulsivity

Wright et al. (9) demonstrated a significant positive association between Factor 1 of the original DIAS, capturing excitability and behavioral control, and motor impulsivity, as indicated by the frequency of paw presses. In contrast, cognitive impulsivity, assessed through maximum delay tolerance, showed a negative association with the same factor. Together, these results imply that motor impulsivity may have a stronger influence on arousal and behavioral regulation, highlighting the importance of accounting for both forms of impulsivity when evaluating dogs' impulsive behaviors. Such dimensions cannot be adequately captured through questionnaires alone; therefore, cognitive and behavioral testing paradigms are required to further validate the German version and its 4-factor structure.

### 4.9 Cross species comparisons: dogs, humans, and ADHD

In humans, impulsivity is typically defined as a predisposition to act without sufficient forethought, often in pursuit of immediate rewards (57). Increasingly, research points to parallels in the manifestation of impulsivity in both humans and dogs, particularly in relation to shared biological mechanisms of self-control and individual neurological differences in cognitive regulation (58–60).

Behaviors in dogs resembling symptoms of attention deficit hyperactivity disorder (ADHD) have also been linked to impulsivity. Vas et al. (20) adapted a questionnaire originally designed to assess ADHD in children to measure inattention and hyperactivity/impulsivity in dogs. More recently, Csibra et al. (61) validated this instrument in a sample of 300 dogs, confirming its potential as a behavioral assessment tool for ADHD-like tendencies in dogs—though not yet appropriate for clinical diagnosis.

The notion that dogs and humans share similar personality traits is further supported by Kubinyi et al. (62), who argued that convergent evolution may have shaped comparable behavioral tendencies in both species. This perspective is reinforced by findings across a broad range of mammals indicating a consistent relationship between impulsivity and aggression (2, 63, 64).

Salonen et al. (56) similarly proposed that dogs may serve as effective models for investigating human psychiatric disorders due to notable overlaps in personality structures. These cross-species similarities suggest that behavioral assessment tools such as the DIAS should be validated in different languages so that they can be used for translational research and across language barriers in the future.

### 4.10 Sociodemographic differences of dogs in the study populations

Regarding the sociodemographic characteristics of dogs, the canine populations in German speaking and Anglo-American regions can be considered comparable. Both the original study and the current study found a higher proportion of pedigree dogs (original study: 76.2%; current study: 59.8%) compared to mixed breed dogs (original study: 23.8%; current study: 39.0%). In both studies, breeds such as Labrador Retrievers, Border Collies, German Shepherds, Golden Retrievers, Jack Russell Terriers, and Poodles were among the 10 most common breeds, further supporting the comparability between German- and English-speaking dog populations. Other breeds frequently reported in the DIAS publication, such as Dalmatians and Shetland Sheepdogs, also appeared among the 20 most common breeds in the current study. However, Cocker Spaniels and Staffordshire Bull Terriers were only minimally represented.

In terms of size categories, the populations also appear comparable. Medium sized dogs (10–25 kg) were most common in both studies (original study: 49.8%; current study: 45.9%), followed by large dogs (25–45 kg; original study: 27.5%; current study: 33.5%) and small dogs (5–10 kg; original study: 18.0%; current study: 14.4%). Very large dogs (> 45 kg) were slightly more common than very small dogs (< 5 kg) in the original DIAS study (2.8 vs. 1.9%), whereas in the current study, both weight categories were equally represented (3.1%).

The sex distribution was roughly balanced in both studies (original study: 52.9% male, 47.1% female; current study: 48.9% male, 50.9% female). However, a higher proportion of neutered dogs was reported in the original study (67.6%) compared to the current study (55.7%).

In summary, the comparison of dog sociodemographic in German speaking and Anglo-American regions shows strong similarities. Both populations are predominantly composed of pedigree dogs, with commonly represented breeds. The distribution of size categories is also comparable, with medium-sized dogs being the most frequent, followed by large and small dogs. Gender ratios are balanced, though neutering was more frequent in the original sample. Dogs in both studies mainly originated from breeders followed by shelters. Overall, the findings support the demographic comparability of canine populations across the two language regions.

### 4.11 Study limitations

Although this study provides valuable insights and strengths, there are some limitations to be acknowledged.

Despite the reasonable large sample size, the predominance of female respondents should be noted, which may limit the generalizability of the results given gender imbalance.

Also, measurement invariance has not been formally tested across relevant subgroups. Consequently, the equivalence of construct measurement across these groups was not conclusively demonstrated and comparison between groups should be approached with caution.

A substantial limitation of this study could be given to the fact that all data rely on caretaker reported information, which may be subject to reporting bias such as social reliability bias potentially leading caretakers to provide answers they perceive to be more socially acceptable or self-selection bias as the participation in this study was voluntary. Also, differences in understanding the questionnaire items and caretakers' perceptions should be considered when interpreting the results.

As test-retest reliability was not re-evaluated in this study it remains unclear whether repeating the questionnaire would have provided similar answers by the participants.

As already stated, the present study is based exclusively on caretaker reported questionnaire data and does not include behavioral testing, physiological measures, biomarkers, or other forms of external validation. Consequently, the factor structure identified should be interpreted as latent behavioral constructs derived from caretaker assessments rather than as directly demonstrated biological entities. Although the emergence of a factor reflecting reactive aggressive behavior is of importance, this factor comprised only two items and should therefore be interpreted with caution. Two-item factors may be less stable psychometrically and can also reflect item-specific covariance rather than a robust latent construct. Consequently, the present findings should be considered preliminary and require replication in independent populations before firm conclusions can be drawn regarding the existence of a distinct impulsivity related dimension.

### 4.12 Future directions

As the primary objective of the present study was the translation and validation of the DIAS into German, future research should aim to systematically investigate additional factors that may influence canine impulsivity. In this context, both dog-specific and caretaker-related sociodemographic variables may provide a valuable foundation for further analyses. Longitudinal study designs, as well as cross-cultural comparative approaches, would be particularly informative in elucidating the developmental trajectories, temporal stability, and contextual variability of impulsivity traits across diverse environments and cultural settings. Furthermore, future research should examine the potential impact of elevated impulsivity on the human–dog relationship and on overall canine welfare.

Given that approximately one third of caretakers in the present sample reported pre-existing behavioral problems in their dogs, it would be advantageous for future studies to analyze this subgroup separately to determine whether impulsivity profiles differ systematically between dogs with and without behavioral concerns. Although test–retest reliability was not assessed in the current study—due to its prior establishment in the original validation of the instrument—subsequent research should seek to replicate this assessment for the German version of the DIAS, thereby further strengthening the evidence base for its psychometric robustness.

## 5 Conclusion

This study demonstrated that the DIAS questionnaire represents a valid instrument for assessing impulsivity-related personality traits in dogs within German-speaking populations, providing evidence for both content and construct validity. The retained factor structure was supported by factor analytic results and allows meaningful interpretation in behavioral terms. The successful translation and validation of the DIAS therefore provide a reliable tool for future assessment and research on canine impulsivity in German-speaking contexts.

## Data Availability

The original contributions presented in the study are included in the article/Supplementary material, further inquiries can be directed to the corresponding author.
