# Supplementary material for: Measuring canine impulsivity: translation and validation of a German dog impulsivity assessment scale
Source: Front Vet Sci. 2026 Aug 6;13:1869578. doi: 10.3389/fvets.2026.1869578 (PMC13491426; doi:10.3389/fvets.2026.1869578)
Supplement: Supplementary file 1 [file Table_1.docx]

**TABLE S1** **Item wording in the original english DIAS version and the translated german version.**

| Item | english wording | german wording |
| --- | --- | --- |
| (1) | My dog shows extreme physical signs when excited (e.g., drooling, panting, raising hackles, urination, licking lips, widening of eyes) | Mein Hund zeigt starke körperliche Zeichen, wenn er/sie aufgeregt ist (z. B. speicheln, hecheln, aufgestellte Rückenhaare, Harnabsatz, Lippen lecken, vergrößerte Augen). |
| (2) | When my dog gets very excited it can lead to fixed repetitive behavior (i.e., an action that is repeated in the same way over and over again), such as tail chasing or spinning around in circles | Wenn mein Hund sich sehr aufregt, kann dies zu einem bestimmten repetitiven Verhalten führen (z. B. sich im Kreis drehen oder den eigenen Schwanz jagen). |
| (3) | I would consider my dog to be very impulsive (i.e., has sudden, strong urges to act; acts without forethought; acts without considering effects of actions) | Ich schätze meinen Hund als sehr impulsiv ein (z. B. hat einen schnell auftretenden starken Drang zu agieren, reagiert ohne nachzudenken, agiert ohne die Auswirkung der Handlung zu berücksichtigen). |
| (4) | My dog doesn't like to be approached or hugged | Mein Hund mag es nicht, wenn auf ihn/sie zugegangen wird oder er/sie umarmt wird. |
| (5) | My dog becomes aggressive (e.g., growl, snarl, snap, bite) when excited | Mein Hund zeigt aggressives Verhalten, wenn er/sie erregt ist (z. B. knurren, schnappen, beißen). |
| *(6)* | *My dog appears to be 'sorry' after it has done something wrong* | *Mein Hund wirkt, als tue es ihm/ihr leid, nachdem er/sie etwas falsch gemacht hat.* |
| (7) | My dog does not think before it acts (e.g., would steal food without first looking to see if someone is watching) | Mein Hund denkt nicht darüber nach, bevor er/sie etwas tut (z. B. würde Futter stehlen, ohne sich vorher zu vergewissern, dass er/sie nicht beobachtet wird). |
| (8) | My dog can be very persistent (e.g., will continue to do something even if it knows it will get punished or told off) | Mein Hund kann sehr beharrlich sein (im Allgemeinen würde er/sie weitermachen, etwas zu tun, auch wenn er/sie weiß, dass er/sie dafür bestraft/gescholten wird). |
| (9) | My dog may become aggressive (e.g., growl, snarl, snap, bite) if frustrated with something | Mein Hund kann aggressiv werden (z. B. knurren, Zähne fletschen, schnappen, beißen), wenn ihn/sie etwas frustriert. |
| (10) | My dog is easy to train | Mein Hund ist leicht zu trainieren. |
| (11) | My dog is not keen to go into new situations | Mein Hund ist nicht begeistert, sich in neue Situationen zu begeben. |
| *(12)* | *My dog takes a long time to lose interest in new things* | *Mein Hund braucht lange, um Interesse an neuen Dingen zu verlieren.* |
| (13) | My dog calms down very quickly after being excited | Mein Hund beruhigt sich sehr schnell wieder, nachdem er/sie sich aufgeregt hat. |
| (14) | My dog appears to have a lot of control over how it responds | Mein Hund wirkt, als ob er/sie viel Kontrolle darüber hat, wie er/sie reagiert. |
| (15) | My dog is very interested in new things and new places | Mein Hund zeigt sich sehr interessiert an neuen Dingen und an neuen Orten. |
| *(16)* | *My dog reacts very quickly* | *Mein Hund reagiert sehr schnell.* |
| (17) | My dog is not very patient (e.g., gets agitated waiting for its food, or waiting to go out for a walk) | Mein Hund ist nicht sehr geduldig (im Allgemeinen erregt, wenn er/sie auf Futter oder einen Spaziergang warten muss). |
| (18) | My dog seems to get excited for no reason | Mein Hund scheint sich unbegründet aufzuregen. |

*Italicized* items (6, 12, 16) represent items without adequate factor loading stability. Item 12 was excluded from the German version of the DIAS (Dog Impulsivity Assessment Scale).
